# DKK1 is a potential novel mediator of cisplatin-refractoriness in non-small cell lung cancer cell lines

**DOI:** 10.1186/s12885-015-1635-9

**Published:** 2015-09-09

**Authors:** Hogir Salim, Dali Zong, Petra Hååg, Metka Novak, Birgitta Mörk, Rolf Lewensohn, Lovisa Lundholm, Kristina Viktorsson

**Affiliations:** Karolinska Biomics Center, Department of Oncology-Pathology, Karolinska Institutet, SE-171 76 Stockholm, Sweden

## Abstract

**Background:**

Platinum compounds are the mainstay of chemotherapy for lung cancer. Unfortunately treatment failure remains a critical issue since about 60 % of all non-small cell lung cancer (NSCLC) patients display intrinsic platinum resistance.

**Methods:**

We analyzed global gene expression profiles of NSCLC clones surviving a pulse treatment with cisplatin and mapped deregulated signaling networks *in silico* by Ingenuity Pathway Analysis (IPA). Further validation was done using siRNA.

**Results:**

The pooled cisplatin-surviving NSCLC clones from each of the biological replicates demonstrated heterogeneous gene expression patterns both in terms of the number and the identity of the altered genes. Genes involved in Wnt signaling pathway (Dickkopf-1, *DKK1*), DNA repair machinery (*XRCC2*) and cell-cell/cell-matrix interaction (*FMN1*, *LGALS9*) were among the top deregulated genes by microarray in these replicates and were validated by q-RT-PCR. We focused on *DKK1* which previously was reported to be overexpressed in NSCLC patients. IPA network analysis revealed coordinate up-regulation of several *DKK1* transcriptional regulators (*TCF4*, *EZH2*, *DNAJB6* and *HDAC2*) in cisplatin-surviving clones from that biological replicate. Knockdown of *DKK1* by siRNA sensitized for cisplatin in two different NSCLC cell lines and in ovarian A2780 cells, but not in the A2780 cis subline made resistant to cisplatin by chronic exposure, suggesting a role of *DKK1* in intrinsic but not acquired platinum refractoriness.

**Conclusions:**

We identified *DKK1* as a possible marker of a cisplatin-refractory phenotype and as a potential novel therapeutic target to improve platinum response of NSCLC cells.

**Electronic supplementary material:**

The online version of this article (doi:10.1186/s12885-015-1635-9) contains supplementary material, which is available to authorized users.

## Background

Lung cancer (LC) is the tumor type with the highest number of cancer-associated deaths worldwide [1]. LC is histologically categorized into non-small cell lung cancer (NSCLC) and small cell lung cancer (SCLC) of which NSCLC constitutes about 85 % of all cases and is further divided into adeno-, squamous cell- and large cell carcinoma [1]. Surgery, if possible, is the treatment of choice for stage I, II and IIIa NSCLC with chemotherapy primarily being used as adjuvant or neoadjuvant treatment [2]. For non-resectable or advanced NSCLC, which constitutes the majority of cases, multimodal chemotherapy alone or in combination with radiotherapy is the main treatment option [2]. The chemotherapy regimen usually consists of a cisplatin or a carboplatin doublet combined with gemcitabine, vinorelbine, paclitaxel, pemetrexed or docetaxel [2]. The primary mechanism of cisplatin action at clinically relevant doses is to induce DNA damage. This is achieved through covalent crosslinking of platinum to the cellular DNA, leading to the formation of crosslinks in the same DNA strand (intra-strand crosslink) or between the two different strands, so called inter-strand crosslinks, ICLs [3]. Subsequently, the ICLs physically impede the progress of the replication fork and transcriptional machinery causing replication stress and blocked transcription process, leading to activation of the intra-S checkpoint, and if the lesions are too extensive, induction of cell death [3].

Cisplatin resistance is still a major obstacle for the clinical management of NSCLC. At the molecular level, a cisplatin-refractory phenotype can be a result of: (I) failure to reach the DNA (pre-target resistance), (II) impeded induction of DNA lesions (on-target resistance), (III) malfunctioning of cell death pathways (post-target resistance), and (IV) activation of pro-survival signaling pathways that are not directly influenced by cisplatin, but abolish its death-inducing capacity (off-target resistance), reviewed in [4].

Although the molecular mechanisms underlying cisplatin refractoriness have been investigated for over a decade, only two biomarkers that can predict cisplatin sensitivity and distinguish responders from non-responders have reached the clinic, excision repair cross-complementing rodent repair deficiency, complementation group 1 (*ERCC1*) and ribonucleotide reductase M1 (*RRM1*), respectively. NSCLC cases whose specimen lacked ERCC1 expression had a more prominent response to adjuvant cisplatin treatment and hence ERCC1 expression holds promise as a predictive biomarker. [5]. Low RRM1 mRNA expression was linked to a better response to a cisplatin/gemcitabine regimen [6]. However, neither *ERCC1* nor *RRM1* were correlated to cisplatin sensitivity when basal mRNA expression was analyzed in 12 NSCLC cell lines [7] reflecting the complexity in finding biomarkers which can predict cisplatin responsiveness.

Other studies have aimed to characterize signaling cascades which could drive cisplatin-survival and hence constitute putative resistance-driving networks in lung cancer by focusing on short term effects of continuous cisplatin treatment i.e. from hours up to a few days, or by creating resistant sub-lines after repeated cisplatin pressure which also could generate new driving mutations [4, 8]. In this study, we explored the intrinsic properties of the cisplatin-surviving sub-population of NSCLC cells 9 days after a single one hour-treatment. This treatment regimen was chosen to reflect the short pulse of drug used clinically, where administration time is typically 30 minutes to two hours (http://www.cisplatin.org/treat.htm).

Using this approach, we found a heterogeneous gene expression pattern when analyzing three biological replicates of cisplatin-surviving NSCLC clones. Among the different biological replicates we identified genes in diverse cellular pathways in these cisplatin-survivors e.g. dickkopf-1 (*DKK1*), X-ray repair cross-complementing protein 2 (*XRCC2*), formin 1 (*FMN1*) and lectin, galactoside-binding, soluble 9 (*LGALS9*). Through bioinformatics analysis, we identified *TCF4*, *EZH2*, *DNAJB6* and *HDAC2* as co-regulated, upstream regulators of DKK1, which may form a signaling circuit that enhances the effect of *DKK1* in enabling survival after cisplatin treatment. By siRNA-mediated knockdown of *DKK1* in NSCLC and ovarian cancer cells, the colony forming capacity and/or cell survival upon cisplatin treatment was reduced significantly. In contrast, plasmid-based overexpression of *FMN1* did not clearly increase cisplatin sensitivity of NSCLC cells. Thus our data suggest that *DKK1* should be further explored as a potential biomarker of cisplatin refractoriness and/or as a target for cisplatin-sensitizing strategies in NSCLC and other tumor types.

## Methods

### Cell lines and culture conditions

In the present study human NSCLC cell lines U-1810 and U-1752 (gifts from Uppsala University, Sweden [9]), A549, H23, H125, H157, H661 and H1299 (ATCC, Manassas, VA, USA) were used. Cells were cultured at 37 °C and 5 % CO_2_ in RPMI-1640 medium containing 2 mM L-glutamine, supplemented with 10 % heat-inactivated fetal bovine serum (both from Invitrogen, Stockholm, Sweden). In addition, the human ovarian cancer cell lines A2780 and its cisplatin-resistant subline A2780 cis (Sigma-Aldrich, Stockholm, Sweden) were used and cultured as above. To maintain the cisplatin resistance of A2780 cis cells, 1 μM cisplatin was added to the culture medium every 3^rd^-4^th^ passage. All cell lines used in the study were established and already published on (see above). No ethical permits were therefore required for their use in the current study.

### Colony formation assay of cisplatin-refractory NSCLC clones

NSCLC cells were seeded in duplicate in Cell + culture dishes (Sarstedt, Landskrona, Sweden) at a density of 500 cells/100 mm dish and were after 24 h treated with cisplatin (2.5-20 μM, Hospira Nordic AB, Stockholm, Sweden) for one hour. Cells were rinsed in PBS after treatment and allowed to form colonies over a 9-days period. The resulting colonies were visualized by staining with crystal violet (0.5 % crystal violet in 25 % methanol) or collected for RNA extraction (see below). For clonogenic survival analyses, colonies consisting of at least 50 cells were counted under a light microscope using duplicate plates from three independent experiments. For retreatment experiments, cell colonies were instead trypsinized and pooled, counted and seeded in 96-well plates for MTT or in new Cell + plates for treatment the next day using the same setup as in the first treatment.

### RNA extraction and gene expression analysis

In order to have enough RNA for the gene expression analysis all the surviving clones from each biological replicate were pooled and subjected to total RNA extraction using Trizol (Invitrogen) as described [10]. Cleanup was performed using the RNeasy Mini kit (Qiagen, Sollentuna, Sweden) and RNA quality was analyzed using an Agilent 2100 Bioanalyzer (Agilent, Santa Clara, CA, USA). Analysis of gene expression was performed using Affymetrix® whole transcript GeneChip® Human Gene 1.0 ST arrays (Affymetrix, Santa Clara, CA, USA), which contains probes for 28 869 genes. cDNA was prepared from 500 ng total RNA, labeled and hybridized to arrays using standard protocols (http://www.affymetrix.com/support/technical/product_updates/wt_1_1_assay.affx). Primary array processing was performed using the Affymetrix GeneChip® Command Console® Software (AGCC, version 1.1) and subsequent analysis was conducted using the Affymetrix Expression Console (EC, version 1.1).

Post-acquisition data processing was carried out using previously described methods (http://www.affymetrix.com/estore/browse/level-1-instruments-software-landingpage.jsp?expand=true&parent=35854&category=35919). Briefly, probe logarithmic intensity error estimation (PLIER) was used to enhance probe signals by summarization; perfect match GC composition-based background correction (PM GCBG) was applied for background correction and global median to normalize the signals. For further analysis, genes with signal intensity below 10 after background correction were excluded to avoid taking genes whose alterations are not easily distinguished from noise into subsequent analyses. In addition, genes corresponding to uncharacterized proteins, hypothetical proteins prefixed with the letters LOC, and small nucleolar RNAs (*SNORD*) were also excluded from the analysis since in this study we aimed to focus on well annotated, protein-coding mRNAs. The raw data presented and used in this article is deposited in NCBI's Gene Expression Omnibus (GEO) [11] as described in the Availability of supporting data section. Hierarchical clustering analysis was performed using Partek Genomics Suite version 6.6 (Partek Inc., St. Louis, MO, USA) in which clustering was based on rows and columns using Euclidean distance for row/column dissimilarity and average linkage as row/column method.

### Quantitative real-time PCR (q-RT-PCR)

For the q-RT-PCR validation of gene expression data, 500 ng of the same RNA batch was used as template for cDNA synthesis using Reverse Transcription Reagents with random hexamer primers (Applied Biosystems, Stockholm, Sweden) as previously described [12]. To quantify mRNA expression levels, cDNA, Fast SYBR®Green Master Mix (Applied Biosystems) and the following primers (*DKK1*, forward: CGG GAA TTA CTG CAA AAA TGG AAT ATG TG, reverse: AAG CTT TCA GTG ATG GTT TCC TCA ATT; *XRCC2*, forward: GGC GAT GTG TAG TGC CTT CCA TA, reverse: TTT CTT TCA AGG AAC TTC TAC CTT CAA GTC; *LGALS9*, forward: AGC TCC AGT GGA ACC AGG TTT G, reverse: TCA TTT CCA CTG AAG CCA GTC TGA A; *ERCC1*, forward: CTG CTT GTC CAG GTG GAT GTG AAA, reverse: GAT ACA CAT CTT AGC CAG CTC CTT GAG. *RRM1*, forward: CCT ATG AGG GCT CTC CAG TTA GCA A, reverse: CCA GTC CCA TAG GTC TGT AGG AGT AAC; *18S*, forward: GCT TAA TTT GAC TCA ACA CGG GA, reverse: AGC TAT CAA TCT GTC AAT CCT GTC C) (from DNA technology, Risskov, Denmark) or *FMN1* (cat.# QT01330315, Qiagen) were mixed in a final volume of 10 μl. The Fast PCR program was used on the ABI Prism 7900HT Sequence detection system (Applied Biosystems), which is initiated at 95 °C for 20 s, followed by 45 amplification cycles (95 °C, 1 s; 60 °C, 20 s). For each biological sample two technical replicates were used and the relative RNA expression obtained by applying the 2^−ΔΔCt^ method [13] in which 18S rRNA was used as an internal control.

### Immunoblotting

Proteins were extracted using RIPA buffer containing 50 mM Tris-HCl, pH 7.4, 150 mM NaCl, 1 mM EDTA, 0.1 % Na-deoxycholate and 1 % NP-40. Thirty microgram of total protein was loaded onto ready-to-use 4-12 % Bis-Tris gels (NuPAGE, Invitrogen), separated by electrophoresis and thereafter blotted onto nitrocellulose membrane (Trans-Blot, Bio-Rad, Hercules, CA, USA). After blocking in Odyssey blocking buffer, diluted 1:1 with TBST (LI-COR Biosciences, Lincoln, NE, USA), primary antibodies recognizing phosphoserine 9 GSK3B, phosphoserine 473 AKT, total AKT and PI3-kinase (5558, 9271, 4685 and 4257, respectively, Cell Signaling Technology, Danvers, MA, USA), p21^WAF1/Cip1^ or Bcl-2 (sc-756 and sc-509, Santa Cruz Biotechnology, Dallas, TX, USA) was added. To control for loading differences, GAPDH (ab9484, Abcam, Cambridge, UK) or β-tubulin (Sigma-Aldrich) was used. To visualize primary antibody binding on the membranes, secondary goat-anti-mouse or goat-anti-rabbit antibodies directly conjugated to infrared dyes, IRDye (LI-COR Biosciences) were applied and resulting protein expression levels analyzed by the Odyssey®Sa Infrared Imaging System (LI-COR Biosciences).

### Ingenuity Pathway Analysis

Ingenuity Pathway Analysis tool (IPA; Ingenuity Systems, Redwood city, CA) was used to create *in silico* interaction networks of *DKK1* based on published, publically available data, showing direct upstream transcription regulators of *DKK1* as well as proteins downstream of *DKK1*.

### MTT cell viability assay

To assess cytotoxic response of cisplatin, MTT (3-[4,5-dimethylthiazol-2-yl]-2,5-diphenyl-tetrazolium) cell viability assay was used in a 96-well format as previously described [14]. Three technical replicates were made for each biological sample and assayed after a continuous exposure to cisplatin for 72 h. For NSCLC cells, 5 000 cells/well were used and in A2780 and A2780 cis experiments, 15 000 cells were seeded per well. Cell viability was assessed by adding the MTT reagent as indicated [14] and is given as % of untreated cells whose viability was set to 100 %. For the NSCLC cells, cisplatin sensitivity was calculated using the area under curve (AUC) from the survival curve.

### DKK1 siRNA transfection

To inhibit *DKK1* expression in U-1810, A549 and A2780/A2780 cis cells, 50 nM siRNA against *DKK1* (si1 = s22721: Sense: GCU UCA CAC UUG UCA GAG Att, Antisense: UCU CUG ACA AGU GUG AAG Cct; si2 = s22722: Sense: GGC UCU CAU GGA CUA GAA Att, Antisense: UUU CUA GUC CAU GAG AGC Ctt, Invitrogen) or non-targeting siRNA (NT, 4390843, Invitrogen) was added to the cells during 72 h (U-1810, A549) or 96 h (A2780, A2780 cis) using Dharmafect 1 (0.1 %) from Dharmacon (Thermo Scientific, Lafayette, CO, USA). Cells were subsequently detached and frozen for RNA extraction or were re-plated for cell death and signaling profiling analysis (collected 24-72 h after cisplatin exposure), for MTT or for colony formation capacity after cisplatin treatment.

#### Overexpression of *FMN1* and assessment of cisplatin sensitivity

*FMN1* was overexpressed in U-1810 cells by transfecting cells with the *FMN1* open reading frame cDNA integrated in the pCMV6-AC-GFP plasmid (OriGene, Rockville, Maryland, USA), using Lipofectamine LTX reagent (Invitrogen, Germany). Briefly, U1810 cells were seeded in 6-well plates and transfected with 2 μg of pCMV6-AC-GFP *FMN1* plasmid for 24 h. As a control, cells only treated with Lipofectamine were used. The next day, media was removed, and normal growth media (RPMI-1640) was added to each well for another 24 h. Western blot analysis was used to confirm the overexpression of FMN1 at the point of cisplatin treatment using a FMN1 antibody (Abcam, Cambridge, UK). To assess the effect on proliferation and cisplatin sensitivity, cells were seeded in 96-well plates (8000 cells/well), and the next day treated with indicated concentrations of cisplatin for 72 h. The cytotoxicity of cisplatin was determined with (3-[4,5-dimethylthiazol-2-yl]-2,5-diphenyl-tetrazolium) (MTT) assay as described above. Survival of cells is given by comparing the absorbance in treated cells relative to the absorbance in cells only treated with Lipofectamine. Three separate transfections were performed with triplicate technical repeats in the MTT. Data presented is the mean ± SEM.

### Statistical analysis

Data given is the mean ± S.D. from three separate experiments, unless otherwise indicated. A two-tailed unpaired Student’s t-test was used. *P*<0.05 was considered for statistical significance.

## Results

### Cisplatin-refractory NSCLC surviving clones show a heterogeneous gene expression pattern

**Fig. 1:**
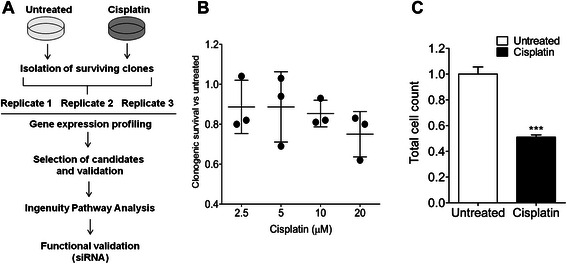
Colony formation assay after treatment of U-1810 cells with cisplatin. **a** Schematic outline of the experiments. NSCLC U-1810 cells were either untreated or cisplatin-treated for 1 h. Cells were then allowed to form colonies over 9 days in drug-free media. One set of dishes was stained with 0.5 % crystal violet and the number of colonies was counted. Other sets of dishes were used for extraction of total RNA for gene expression profiling. Ingenuity Pathway Analysis software was used to identify altered cellular networks based on differential gene expression (>1.5 fold up- or down-regulated) relative to untreated cells. A number of genes were selected for further validation by q-RT-PCR. **b** Relative clonogenic survival of NSCLC U-1810 cells after treatment with 2.5-20 μM cisplatin. For each concentration, three biological replicates were performed where the survival of untreated cells was set to one. **c** Total cell number was calculated using a haemocytometer for the surviving clones after 10 μM cisplatin. ***; *p*<0.005

In order to identify underlying signaling aberrations of the NSCLC cells which could govern a cisplatin-refractory phenotype, the gene expression pattern of long-term cisplatin-surviving NSCLC clones was analyzed in three biological replicates and compared with that of untreated cells which formed colonies, as outlined in Fig. 1a. First, cytotoxic profiling of NSCLC U-1810 cells after cisplatin treatment was carried out using concentrations in the range achievable in plasma from patients (5 μM) [15] (Fig. 1b). The U-1810 cells displayed a clear cisplatin-refractory phenotype as 2.5, 5 and 10 μM cisplatin only reduced the colony forming capacity by 10 %, while at 20 μM cisplatin, the reduction was 20 % (*p* = 0.02) (Fig. 1b). Although the difference in clonogenicity was minor, 10 μM cisplatin caused a 2-fold decrease in total cell number (Fig. 1c). This demonstrates that at therapeutically relevant concentrations, the reduction in growth rate is more pronounced than the effects on colony number after platinum treatment.

Next we pooled the surviving NSCLC clones from untreated or cisplatin-treated cells within each biological replicate and performed gene expression array analysis. First, we sought to identify genes which were consistently altered in all three biological replicates examined using a cutoff value of 1.5-fold up- or down-regulated. From this analysis only one gene, formin 1 (*FMN1*), previously reported to control cell morphology by regulating focal adhesion and motility [16], was regulated in all replicates. Despite similar treatment conditions in the three biological replicates of cells, yet they showed some differences in cisplatin response (Fig. 1b).

We reasoned that as cisplatin may confer resistance in multiple ways, the heterogeneity among the biological replicates could possibly reflect a biologically heterogeneous response but it cannot be ruled out that also experimental variations by other means could contribute to the observed results. As a next step we therefore analyzed alterations in gene expression in each biological replicate separately. In total, 2720 genes were up- and 2725 genes were down-regulated in the cisplatin-surviving clones from the first replicate, while 1238 and 50 genes were up- and 46 and 84 genes were down-regulated in the surviving clones from the second and third replicates, respectively (Table 1). Importantly, the difference in direction of regulation, magnitude as well as in the number of altered genes among the three biological replicates varied (Additional file 1, Table 1). This suggests that there is indeed considerable heterogeneity among the surviving NSCLC clones on the transcriptome level, which also was evident by hierarchical clustering (Additional file 1).

**Table 1:** Number of regulated genes in cisplatin-surviving clones

	No. of genes (≥1.5-fold)	
	Up-regulated genes	Down-regulated genes
Replicate 1	2720	2725
Replicate 2	1238	46
Replicate 3	50	84
Replicate 1 + 2	19	8
Replicate 1 + 3	9	11
Replicate 2 + 3	7	0
Replicate 1 + 2 + 3	0	1

The numbers of differentially expressed genes in each replicate of the NSCLC residual U-1810 clones after cisplatin treatment.

**Fig. 2:**
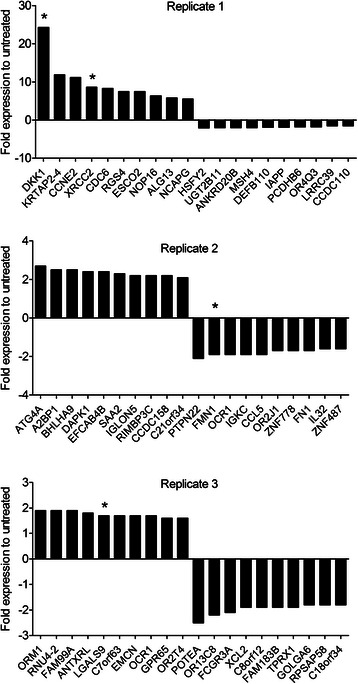
Cisplatin-surviving NSCLC clones have heterogeneous gene expression patterns. Global transcriptome analysis was performed on RNA extracted from cisplatin-surviving (1 h, 10 μM) versus untreated NSCLC clones. Fold changes in the expression (relative to untreated sample) of the top 10 up- and down-regulated genes from each biological replicate are depicted separately; replicate 1 (upper panel), replicate 2 (middle panel), replicate 3 (lower panel). Stars mark candidate genes that were subsequently validated by q-RT-PCR

By analyzing each biological replicate separately a number of genes with altered expression in cisplatin-surviving NSCLC clones as compared to untreated counterparts were evident. Among the top 10 regulated genes from the cisplatin-refractory NSCLC clones were dickkopf-1 (*DKK1*) and X-ray repair cross-complementing protein 2 (*XRCC2*) from the first replicate, and lectin, galactoside-binding, soluble 9 (*LGALS9*) from the third replicate (Fig. 2, labeled with *). These proteins have been implicated in the prognosis of different tumors [17–19]. DKK1 is a secreted protein that inhibits bone formation through inhibition of Wnt signaling pathway and has been shown to be highly expressed in NSCLC tumor material and serum from NSCLC patients as compared to patients with no tumor [18, 20]. DKK1 has also been shown to promote invasion and migration in NSCLC cells *in vitro* [21]. In our gene expression analysis, *DKK1* was 24-fold higher expressed in the cisplatin-refractory clones than in the untreated clones in that biological replicate (Fig. 2, top panel).

In accordance with other studies which demonstrated a role of homologous recombination (HR) in inter-strand crosslink tolerance [19], we observed an 8-fold up-regulation of *XRCC2* in cisplatin-refractory NSCLC clones. Finally, in the third biological replicate *LGALS9* expression was 1.7-fold higher in residual NSCLC clones than in untreated clones (Fig. 2, lower panel). *LGALS9* is a member of beta-galactoside-binding proteins (galectins) and has been described to be a negative regulator of the antitumor immune T cells [17]. Importantly, although we found that each of these genes were up-regulated in different biological replicates of cisplatin-refractory NSCLC residual clones, their expression levels were largely unchanged in the other replicates further illustrating that the NSCLC residual clones that survive cisplatin pulse treatment have heterogeneous gene expression patterns.

### Validation of altered FMN1, DKK1, XRCC2 and LGALS9 mRNA expression in cisplatin-refractory NSCLC residuals

**Fig. 3:**
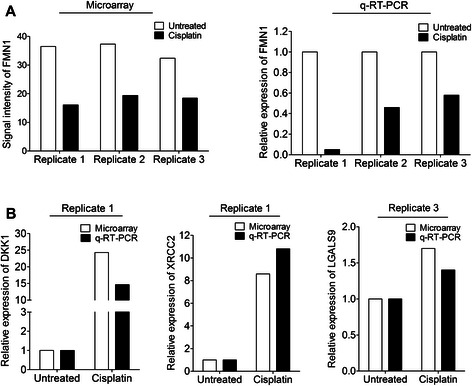
Validation of potential markers for intrinsic cisplatin refractoriness by q-RT-PCR. **a** Expression levels of *FMN1* in cisplatin-surviving clones from all three biological replicates were determined by microarray analysis (left panel) and q-RT-PCR (right panel). **b** Relative mRNA expression of *DKK1*, *XRCC2* and *LGALS9* was determined by q-RT-PCR from replicates 1 and 3, respectively. For all mRNA analyses, 18S rRNA was used to control for loading differences among the samples. The relative expression of each gene in cisplatin-surviving NSCLC residual clones is given as fold change relative to untreated NSCLC clones which are set to one

Next, we set out to validate the observed alterations in *FMN1*, *DKK1*, *XRCC2* and *LGALS9* expression by q-RT-PCR using the same RNA as was applied in the microarray analysis (Fig. 3). In the global transcriptome analysis, *FMN1* expression was reduced in the cisplatin-surviving NSCLC residual clones by about 50 % (Fig. 3a, left panel). Accordingly, q-RT-PCR revealed a similar down regulation of *FMN1* mRNA expression in all three replicates confirming the observed alteration of this gene in cisplatin-refractory clones (Fig. 3a, right panel). We also tested if overexpression of *FMN1* in NSCLC U-1810 cells could sensitize for cisplatin treatment (Additional file 2). Albeit a clear overexpression of FMN1 was achieved (Additional file 2A) and *FMN1* alone caused a slight decrease in cell viability, no statistically significant effect on cisplatin response was evident (Additional file 2B)*.* Hence this data suggest that the observed down regulation of *FMN1* in cisplatin refractory clones is not directly associated with resistance, or acts in concert with other signaling components in order to regulate cisplatin responsiveness.

*DKK1*, the top scored gene in the first biological replicate showed a 25-fold increase in the cisplatin-refractory clones by global gene expression profiling and a 15-fold up-regulation by q-RT-PCR analysis (Fig. 3b). In addition, *XRCC2* expression was increased about 10-fold when analyzed by either microarray or q-RT-PCR, in replicate 1 (Fig. 3b). Finally, *LGALS9* expression was 1.7-fold higher in NSCLC cisplatin-refractory clones as analyzed by microarray and similarly 1.4-fold higher than untreated cells by q-RT-PCR in replicate 3 (Fig. 3b). In conclusion, we were able to validate the altered expression of *FMN1*, *DKK1*, *XRCC2* and *LGALS9* observed with gene expression profiling.

### DKK1 pathway proteins show concurrent up-regulation in cisplatin-refractory NSCLC clones

**Fig. 4:**
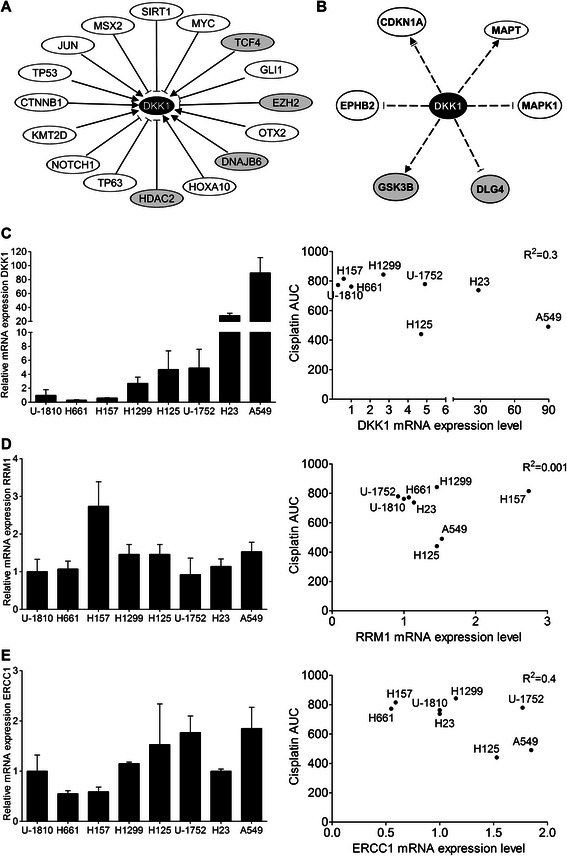
*In silico* mapping of the *DKK1* interactome using Ingenuity Pathway Analysis (IPA). **a** IPA-generated network built around *DKK1* showing the direct upstream transcriptional regulators. Those that showed concurrent up-regulation along with *DKK1* in replicate 1 are shown in grey. **b** IPA-generated network centered on *DKK1* showing downstream signaling proteins that are indirectly regulated by *DKK1*. Those that were regulated also in replicate 1 are shown in grey. For **a** and **b**, activation (►), inhibition (┤) or reports on both (┤►), indicates the regulation retrieved from Ingenuity. **c** Left panel: Q-RT-PCR showing the mRNA expression level of *DKK1* in a panel of NSCLC cell lines. 18S rRNA was used as a loading control. Right panel: Correlation between baseline *DKK1* mRNA expression and the cisplatin sensitivity of NSCLC cell lines after prolonged exposure (measured as area under the curve (AUC) in MTT assay after 72 h continuous treatment, based on three experiments each performed in triplicate). mRNA expression levels of *RRM1* **d** and *ERCC1* **e** together with correlation to cisplatin sensitivity as described in **c**

To further explore the *DKK1* pathway in the cisplatin-refractory phenotype of NSCLC cells and to delineate putative mechanisms, Ingenuity Pathway Analysis (IPA) was used to map upstream regulating and downstream proteins of *DKK1* (Fig. 4). First, an interaction network based on published literature composed of proteins regulating *DKK1* was created and from this the transcriptional regulators of *DKK1* were selected for further analysis (Fig. 4a). In total, IPA identified 16 transcriptional regulators of *DKK1* out of which 4 showed a concurrent up-regulation in the NSCLC cisplatin-surviving clones (Fig. 4a). Thus the expression of transcription factor 4 (*TCF4*), enhancer of zeste homolog 2 (*EZH2*), DnaJ homolog subfamily B member 6 (*DNAJB6*) and histone deacetylase 2 (*HDAC2*) showed 2.0-, 3.0-, 1.6- and 2.3-fold increase, respectively, in the cisplatin refractory NSCLC clones relative to the corresponding untreated clones of replicate 1. The finding that different factors in the *DKK1* pathway are coordinately up-regulated in cisplatin-surviving NSCLC clones may point towards a role for *DKK1* in driving a cisplatin-refractory phenotype.

In order to identify signaling components downstream of *DKK1* which could have a role in the control of a cisplatin-refractory phenotype, a similar network was constructed by IPA. Albeit some direct protein-protein interactions are reported, only indirect targets of DKK1 were available in IPA. Cyclin-dependent kinase inhibitor 1A (*CDKN1A*, also called p21^WAF1/Cip1^) [22, 23]*,* microtubule-associated protein tau (*MAPT*) [24–26], mitogen-activated protein kinase 1 (*MAPK1, ERK2*) [27], disks large homolog 4 (*DLG4*) [28], glycogen synthase kinase 3 beta (*GSK3B*) [28, 29] and ephrin type-B receptor 2 (*EPHB2*) [30] were found to be indirectly regulated by *DKK1* and could therefore potentially be mediators of the DKK1 effect (Fig. 4b). *GSK3B*, which is reported to be a master negative regulator of diverse signaling pathways including Wnt and insulin signaling [31], displayed a 1.5-fold increased expression in replicate 1. *DLG4*, which encodes a neuronal signaling and cell polarity protein with a potential tumor suppressor role [32], was 1.8-fold down-regulated in replicate 1. The other reported *DKK1* downstream proteins displayed only minor changes in mRNA expression in NSCLC cisplatin-refractory clones indicating that these are not downstream targets in this setting (for deposited data, see Availability of supporting data).

### Basal DKK1 expression does not correlate to cisplatin sensitivity

Given the observed up-regulation of *DKK1* in cisplatin-refractory NSCLC residual clones, we next analyzed basal mRNA *DKK1* expression levels in NSCLC cell lines with the aim to reveal if there was a correlation between basal *DKK1* expression level and cisplatin sensitivity (Fig. 4c). A heterogeneous expression level of *DKK1* was evident among the NSCLC cell lines with the highest *DKK1* mRNA expression found in A549 and H23 cells, which displayed an about 80- and 30-fold higher expression than that observed in the U-1810 cells, which were used for the gene expression profiling of residual clones (Fig. 4c, left panel). We next set out to analyze if there was a correlation between basal *DKK1* expression on mRNA level and cisplatin responsiveness. The NSCLC were subjected to 72 h continuous treatment with cisplatin and the area under the curve (AUC) was used as a measurement of cisplatin sensitivity. No correlation between baseline *DKK1* expression and platinum sensitivity was however evident (Fig. 4c, right panel). Of note, the cisplatin sensitivity was relatively similar for the NSCLC cell lines which potentially could explain the lack of correlation between *DKK1* and cisplatin response Moreover, the mRNA levels of the previously published markers of cisplatin resistance, *RRM1* and *ERCC1*, were also analyzed in relation to cisplatin responsiveness (Fig. 4d-e). Their expression was generally much less diverse than *DKK1* in our NSCLC cell line panel (Fig. 4d-e), and not correlated to their platinum sensitivity.

### Ablation of DKK1 expression sensitizes NSCLC cells to cisplatin

**Fig. 5:**
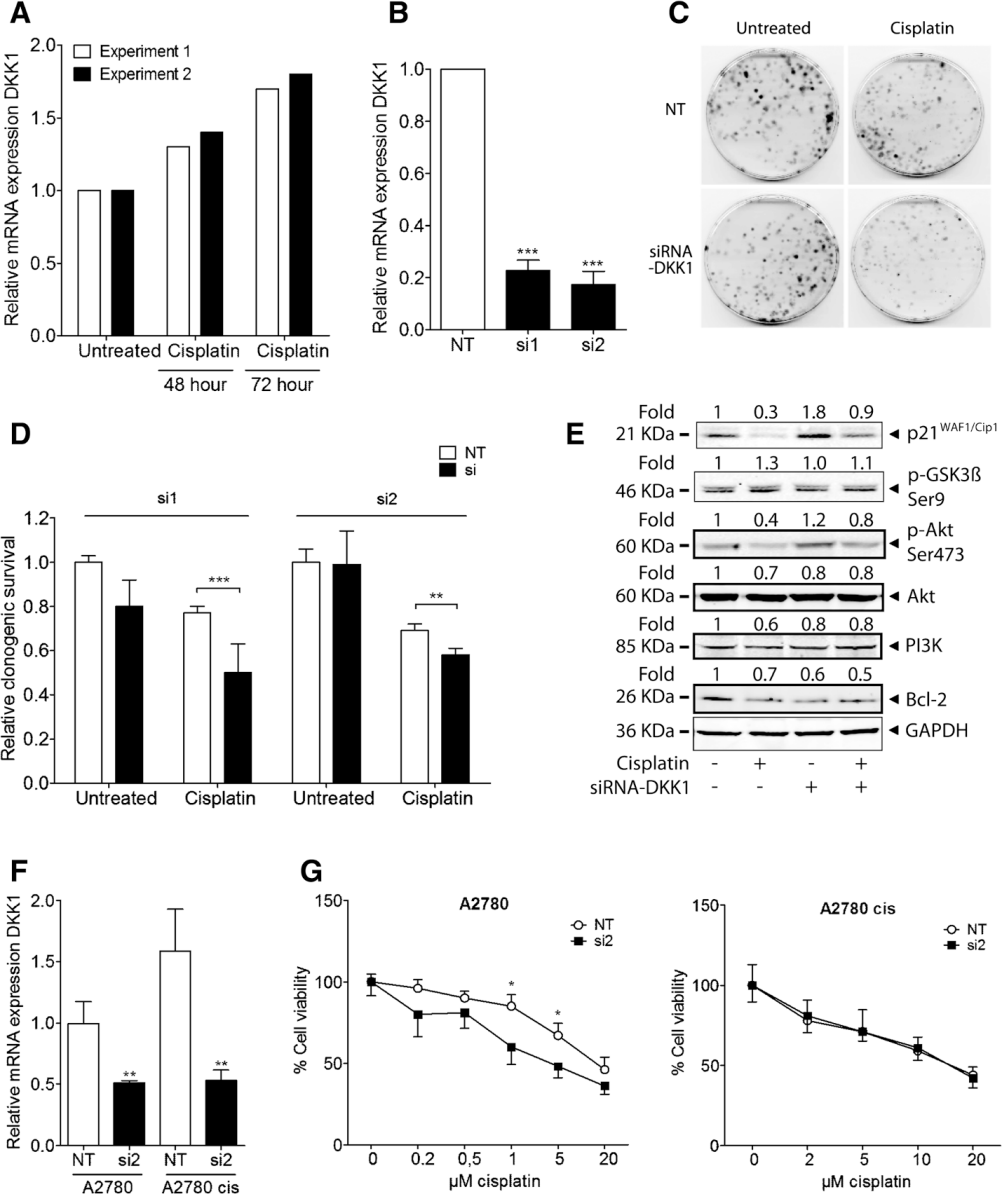
Knockdown of *DKK1* sensitizes NSCLC cells to cisplatin. (**a**) Q-RT-PCR shows the relative mRNA expression of *DKK1* 48 and 72 h after a short pulse treatment of U-1810 cells with 10 μM cisplatin (1 h). The results of two separate experiments are shown. **b** U-1810 cells were transfected with non-targeting (NT) or *DKK1*-specific siRNAs (si1 or si2); silencing of *DKK1* mRNA expression was confirmed by q-RT-PCR. 18S rRNA was used as a loading control. ***; *p*<0.005 vs NT control. **c** U-1810 cells were transfected with siRNA-*DKK1* or NT siRNA and colony formation capacity was assayed 9 days after treatment or not with a short pulse of cisplatin (1 h, 10 μM). Representative images of culture dishes for si1. **d** Clonogenic capacity relative to untreated, NT siRNA-transfected cells for U-1810 si1 and si2**.** ***; *p*<0.005, **; *p*<0.01, when comparing DKK1 siRNA-transfected to NT siRNA-transfected cells after cisplatin treatment. **e** Western blots showing p21^WAF1/Cip1^, phosphorylated GSK3B (Ser9) and AKT (Ser473), total AKT, PI3K and Bcl-2 in U-1810 cells 24 h after treatment with cisplatin (1 h, 10 μM), which was performed on reseeded cells after the 72 h-transfection with NT control or siRNA-*DKK1* (si1). GAPDH was used as a loading control. (**f-g**) A2780 and its cisplatin-resistant subtype A2780 cis cells were transfected with siRNA-*DKK1* (si2) or NT. **f** siRNA silencing of *DKK1* mRNA expression was confirmed by q-RT-PCR. **; *p*<0.01. **g** Cell viability was assayed 72 h after continuous treatment with cisplatin, relative to untreated NT or si, using MTT assay. *; *p*<0.05

In order to functionally connect *DKK1* expression to cisplatin response we next analyzed the effect of cisplatin on *DKK1* expression in NSCLC U-1810 cells at 48 and 72 h after a 1 h pulse treatment with 10 μM cisplatin (Fig. 5a). As shown in Fig. 5a, transient cisplatin exposure led to a slightly (1.3-fold) increased *DKK1* mRNA expression at 48 h post treatment which was further increased to 1.7-fold after 72 h. This may suggest that cisplatin can elicit increased *DKK1* expression as a protective response. To explore if *DKK1* is of importance for cisplatin refractoriness, *DKK1* expression was ablated in NSCLC U-1810 and A549 cells using siRNA and the effect on clonogenic survival was examined. An 80-90 % ablation of *DKK1* mRNA expression was achieved after transfection with either of two different siRNA (si1 and si2) for 72 h as compared to the non-targeting control siRNA in both NSCLC cell lines (Fig. 5b and Additional file 3A). Notably, while cisplatin treatment or knockdown of *DKK1 per se* each reduced the colony formation capacity by up to 30 %, the combination of these treatments reduced colony formation by 50 % in U-1810 cells, demonstrating that *DKK1* ablation sensitizes these NSCLC cells to cisplatin (Fig. 5c-d). Moreover, the same cisplatin-sensitizing effect of *DKK1* knockdown was also evident in A549 cells but at a slightly lower magnitude (Additional file 3B). In summary, these data indicate that knockdown of *DKK1* confers long term cisplatin sensitization in NSCLC cells and results in reduced colony forming capacity.

The influence of *DKK1* ablation on two of its IPA-suggested indirect downstream targets i.e. p21^WAF1/Cip1^ [22, 23] and *GSK3B* [28, 29] was also examined in non-targeting or si*DKK1*-transfected U-1810, 24 h after treatment with cisplatin for 1 h (Fig. 5e). Knockdown of *DKK1 per se* increased the expression of p21^WAF1/Cip1^ (Fig. 5e), implicating G1 arrest and possibly a senescent phenotype. In these p53-lacking U-1810 cells [33], cisplatin generally reduced the p21^WAF1/Cip1^ levels (Fig. 5e), however when si*DKK1* was applied the reduction was smaller and a higher p21^WAF1/Cip1^ expression level remained similar to untreated, non-targeting cells. This was supported in the p53 wild type A549 cells where cisplatin increased p21^WAF1/Cip1^, the increase is however more pronounced after si*DKK1* and cisplatin than after cisplatin alone (Additional file 3C). The activity of GSK3B, which is decreased by phosphorylation at serine 9, was however not significantly affected upon *DKK1* ablation or cisplatin treatment (Fig. 5e).

An increased activity of growth factor-regulated kinases and up-regulated expression of anti-apoptotic proteins are reported to contribute to a cisplatin-refractory phenotype and to hamper cisplatin response in solid tumor cells [4]. Phosphatidylinositol-3-kinase/v-Akt murine thymoma viral oncogene homolog (PI3K/AKT) signaling has been demonstrated to be increased in cisplatin-refractory ovarian and colon cancer cells [34–36]. Moreover, the activation of AKT by Ser473 phosphorylation is reported to in part impair cisplatin-induced apoptosis by inactivating Bcl-2-associated death promoter (BAD) or by increasing B-cell lymphoma 2 (Bcl-2) expression levels, thereby blocking Bcl-2 homologous antagonist/killer/Bcl-2-associated X protein (BAK/BAX) activation [37, 38]. Accordingly, we examined the phosphorylation of AKT (Ser473) and total AKT, PI3K and Bcl-2 expression levels in these si*DKK1*-transfected, cisplatin-treated U-1810 cells (Figure 5e).

The basal level of phospho-AKT (Ser 473) was slightly increased in *DKK1*-knocked cells, while cisplatin reduced the levels in both non-targeting and *DKK1* siRNA-transfected cells. Only minor decreases in total AKT and PI3K expression was seen after cisplatin and/or *DKK1* siRNA (Fig. 5e). Our results suggest that although PI3K/AKT signaling might still be players in this context, they do not seem to play a prominent role in the increased cisplatin sensitivity of cells lacking DKK1. On the other hand, in both U-1810 and A549 cells, Bcl-2 displayed a reduced expression in DKK1-knocked cells compared to non-targeting, untreated cells (Fig. 5e, Additional file 3C). Similarly, cisplatin caused a reduced expression of Bcl-2 and when DKK1 siRNA and cisplatin were combined, down-regulation was clearly evident. The expression of another Bcl-2 family member, B-cell lymphoma-extra large (Bcl-xL), was not altered in these samples (data not shown). Albeit further studies on the role of apoptotic signaling in DKK1 siRNA-mediated signaling to cisplatin is needed, this data suggests that *DKK1* ablation may sensitize for cisplatin by down-regulating Bcl-2 expression.

### DKK1 knockdown sensitizes ovarian cancer cells to cisplatin

To validate our results from NSCLC in another tumor type, we tested the ovarian cancer cell lines A2780 and A2780 cis. A2780 cis is a subline of A2780 that developed acquired cisplatin resistance after exposure to increasing concentrations of cisplatin over time [39]. Hence A2780 cis is about 7-fold more resistant to cisplatin than its parental A2780. When applying DKK1 siRNA (si2), DKK1 mRNA expression was down to about 50 % in the parental A2780 cell line and to approximately 30 % in A2780 cis versus their respective non-targeting control (Fig. 5f). When assaying DKK1 mRNA levels, a higher basal level (1.6-fold) was found in A2780 cis as compared to A2780 cells (Fig. 5f). siDKK1 significantly sensitized the A2780 , but not the A2780 cis cells, to cisplatin treatment (Fig. 5g). In summary, these data indicate that knockdown of DKK1 also can sensitize cells of other tumor types than NSCLC to cisplatin. The acquired resistance of A2780 cis was however more difficult to target and not possible to revert at the level of DKK1 knockdown achieved in these experiments. Nevertheless, A2780 cis had a higher baseline DKK1 expression level which fits with our data of an involvement of DKK1 in cisplatin resistance.

## Discussion

Platinum-based compounds e.g. cisplatin and carboplatin constitute the standard chemotherapy regimen for NSCLC. Unfortunately a large proportion of the cases display intrinsic resistance to these platinum drugs and for yet another fraction, a platinum-refractory phenotype typically develops during the treatment course [40]. In this study, we aimed to identify molecular determinants which drives a cisplatin-refractory phenotype and hence could be used either as biomarkers of response or as sensitizing targets for cisplatin in NSCLC. Our approach of studying gene expression alterations in cisplatin-surviving NSCLC clones is different from previous reports using either very high, non-clinically achievable cisplatin doses in short term treatment schedules [41] or tumor cell models of acquired resistance [42]. The latter is mostly reported to result in resistance mechanisms involving up-regulation of membrane-associated drug efflux pumps such as ATP-binding cassette proteins and copper-extruding P-type ATPases [4, 8].

In our three biological replicates only *FMN1* showed altered expression in all three replicates, illustrating that NSCLC cells surviving cisplatin pulse treatment have heterogeneous clonogenic survival capacity and gene expression patterns. A possible reason may be that few prominent long term effects are seen on the RNA level 9 days after cisplatin treatment. However, one interpretation of this outcome is that cisplatin treatment can result in the expansion of different resistant clones in different experiments. This clonal evolution hypothesis has been demonstrated after epidermal growth factor receptor (*EGFR)*-ablative therapy, where a very low number of Kirsten rat sarcoma viral oncogene homolog (*KRAS)*-mutated colorectal cancer cells emerged to become the dominant clone among the surviving cells [43, 44]. Hence, we speculate that even small initial variations in cisplatin responsiveness can induce certain clones to become dominant. Optimally, if not limited by the minimal amounts needed for the analysis method, it would be interesting to analyze multiples of clones separately to explore their differences and heterogeneity further.

We and others have shown that a chemotherapy-refractory cancer stem cell phenotype is evident in certain NSCLC cell lines [14, 45]. However, this sphere-forming capacity after enrichment in stem cell media was not found in the NSCLC cell line used in this study U-1810, suggesting that they might not contain an appreciable proportion of stem-like cells and that the heterogenic response of chemotherapy in this particular cell line likely is governed by other signaling cascades. We observed the same cisplatin response in clonogenic and MTT assays upon retreatment (Additional file 4), therefore we could verify that using our single-treatment setup, we were most likely only studying the primary effects in the surviving clones that were selected due to intrinsic refractoriness.

The one gene that was down-regulated in all three biological replicates was formin 1 (*FMN1*), a protein which enhances formation of cell-cell adhesion [16]. As cisplatin disrupts cell-cell adhesion before it induces apoptosis [46], one may speculate that the fraction of cells with low *FMN1* expression may be less responsive to the adhesion-disruptive effects of cisplatin, and consequently survive. However, by overexpressing *FMN1* we were not able to sensitize NSCLC cells to cisplatin indicating that either FMN1 is not directly involved in regulating cisplatin sensitivity or it acts in concert with other signaling aberrations to confer survival advantage if down-regulated, which not is recapitulated when forced overexpression is used.

Analysis of each individual experiment revealed *DKK1*, *XRCC2* and *LGALS9* as top scored differentially up-regulated genes in cisplatin-surviving clones from replicates 1 and 3, respectively. It is well documented that cisplatin treatment activates multiple DNA damage signaling cascades, and here we found an increased expression of *XRCC2,* a member of the homologous recombination repair pathway, in cisplatin-refractory residual NSCLC clones. This up-regulation might be due to inherent properties of the cells, or alternatively, a selective pressure on the surviving clones to up-regulate proteins involved in DNA repair to withstand the damage. In line with our data, mouse embryonic fibroblasts deficient in *XRCC2* are reported to be hypersensitive to cisplatin treatment [47], further pointing towards a connection between high *XRCC2* expression levels and cisplatin resistance. Albeit *LGALS9* has not yet been implicated in NSCLC or in a chemotherapy-refractory phenotype of other tumor cells, various galectins such as galectin-1 and -3 were reported to have a role in driving a chemotherapy-refractory phenotype [48, 49].

Importantly, we demonstrate that *DKK1* has a role in the intrinsic platinum responsiveness of NSCLC, as siRNA-mediated ablation of *DKK1* sensitized NSCLC cells to cisplatin and reduced their clonogenic survival potential. *DKK1* is a secreted protein with dual anti- and pro-survival functions in different tumor types. For instance, *DKK1* may act as a tumor suppressor through inhibition of Wnt/β-catenin signaling and is reported to activate apoptosis in multiple tumor types e.g breast cancer, renal cell carcinoma, melanoma and choriocarcinoma [50–53]. In head and neck cancer cells, decreased *DKK1* expression was associated with acquired cisplatin resistance [42], whereas overexpression of *DKK1* in a glioma cell line sensitized these cells to DNA damaging agents including cisplatin [54]. Some of these data are opposed to our study in which DKK1 was upregulated in cisplatin-surviving NSCLC clones and its knockdown conferred cisplatin sensitivity. These differences could possibly be attributed to tumor type specific divergences in signaling cascades, or in mechanisms of acquired cisplatin resistance. Our results of cisplatin sensitization from NSCLC were however validated also in ovarian cancer cells which were sensitized to cisplatin upon siRNA knockdown of *DKK1.* Yet we could not sensitize the acquired cisplatin-resistant subclone A2780 cis at the level of knockdown achieved in our experiments. Our interpretation is that DKK1 regulates intrinsic cisplatin resistance, still it may not be the main driver of acquired cisplatin resistance.

Multiple studies have demonstrated an oncogenic role of *DKK1* in diverse tumor types such as multiple myeloma, hepatoblastoma, Wilm’s tumor and hormone-resistant breast cancer [55–57]. Moreover, high serum level of DKK1 has been detected in patients with NSCLC and esophageal carcinoma where it was associated with tumor progression and poor outcome of these malignancies suggesting that *DKK1* in these tumor malignancies may have an oncogenic role [18, 20, 58]. Using the cBioPortal for Cancer Genomics (cbioportal.org) [59, 60] which integrates data from several databases including The Cancer Genome Atlas, we found that *DKK1* was altered at the level of either mRNA upregulation, mutation, homozygous deletion or amplification in a total of 6-9 % of lung adeno- or squamous cell carcinoma patients [61, 62]. In the adenocarcinoma population, the mentioned alterations in DKK1 were also linked to a significantly reduced overall survival [62], further supporting the importance of DKK1 in NSCLC. Yet it remains to be demonstrated if DKK1 regulate intrinsic cisplatin sensitivity *in vivo*. Such studies could be performed using NSCLC patient-derived xenografts in mice. To demonstrate that DKK1 is a predictor of cisplatin refractoriness *in vivo* in NSCLC patients is more challenging as it would require a biopsy of primary tumor and metastasis prior and post cisplatin treatment which is not a standard routine in our clinic at present. Hence a controlled clinical trial would be required in order to adequately address this issue.

Through bioinformatics analysis of *DKK1*, we identified a number of putative transcription regulators of this gene. Specifically, ectopic expression of the Wnt signaling components TCF4 as well as active β-catenin induce transcription of the *DKK1* gene, and the *DKK1* promoter contains several *TCF4* response elements, which fits well with our data of co-regulated *TCF4* and *DKK1* [63]. *DNAJB6* is known to activate *DKK1* expression and also had an increased expression in our data demonstrating a regulation which fits earlier reported alterations [64]. In contrast, *EZH2* and *HDAC2* which cause repression of *DKK1* according to literature [65–67], also showed increased expression in our data. However, at least the *HDAC2* effects were reported to be p53-dependent [67] and might therefore not apply in this cell system since U-1810 cells lack p53 expression due to a truncating mutation at p53 codon 172 [33]. Nevertheless, additional validation experiments using siRNA/overexpression of these proteins are needed to confirm a role for these transcriptional regulators in the observed increased *DKK1* expression in the cisplatin-refractory NSCLC clones.

IPA analysis identified p21^WAF1/Cip1^ to be a putative downstream effector protein of *DKK1*, and p21^WAF1/Cip1^ is reported to negatively regulate the cell cycle, i.e. to have a tumor suppressor role [68]. In rat mesenchymal stem cells, addition of recombinant DKK1 protein decreased p21^WAF1/Cip1^ mRNA levels as well as the β-gal staining, both indicators of senescence [23]. This is in line with our data where DKK1 knockdown increased p21^WAF1/Cip1^. Another IPA-retrieved report show however that transgenic mice with ectopic expression of DKK1 in intestinal crypts has an up-regulated p21^WAF1/Cip1^, possibly as a consequence of repression of c-myc expression [22]. Overexpression or silencing of *p21*^*WAF1/Cip1*^ induced or reduced, respectively, the cytotoxicity of cisplatin in NSCLC A549 cells, signifying its importance in cisplatin response in NSCLC [69]. After cisplatin treatment, an increased expression of p21^WAF1/Cip1^ is commonly seen in p53 wild type cell lines [70], like we see in A549 cells (Additional file 3C). Although p21^WAF1/Cip1^ was decreased in the p53-mutant U-1810 cells after cisplatin, the level was higher after si*DKK1* combined with cisplatin. Data from A549 cells support this elevated p21^WAF1/Cip1^ level in si*DKK1*-ablated, cisplatin-treated samples, despite their differential response to cisplatin. Therefore we speculate that p21^WAF1/Cip1^ could contribute to the reduced growth after *DKK1* knockdown and cisplatin by induction of G1 arrest and senescence.

GSK3B is a negative regulator of Wnt signaling pathway and inhibition of GSK3B activity, i.e. increased p-Ser9, has previously been shown to confer resistance to cisplatin in lung and ovarian cancer cells [29, 71, 72]. The mRNA expression was co-regulated with DKK1 in the cisplatin-refractory cells but we did not see any change in the phospho-GSK3B at the time point studied after si*DKK1*. Still, the previously reported *DKK1*-regulation (Fig. 4b) of both *GSK3B* (up) and *DLG4* (down) was confirmed in replicate 1.

No major changes were seen when we analyzed the PI3K/AKT proteins which are known to be involved in cisplatin-refractoriness [35]. We did however see an almost 2-fold down-regulation in expression of the anti-apoptotic protein Bcl-2 in both DKK1 siRNA and DKK-1 ablated and cisplatin-treated samples in both U-1810 and A549 cells (Fig. 5e and Additional file 3C). A reduced Bcl-2 allows for activation of pro-apoptotic BAK/BAX, which is required for proper cisplatin response [37], i.e. increased cisplatin-induced apoptosis. This could serve as a mechanism for the sensitization since elevated levels of Bcl-2 and other proteins within the same family e.g. BCL-XL and MCL1 correlate with cisplatin resistance as well as tumor recurrence in NSCLC and other cancers [73–76]. Small molecule inhibitors for Bcl-2-like proteins are also tested in clinical trials together with cisplatin [77]. Yet the importance of this down-regulation and the role of DKK1 in regulating cisplatin-induced apoptotic signaling would require further studies.

Apart from DKK1's role as a Wnt-signaling antagonist, DKK1 overexpression correlates to an accumulation of β-catenin in the cytoplasm or nucleus in clinical samples from hepatocellular carcinoma [78]. We analyzed the total level of β-catenin protein (data not shown) in the samples from Fig. 5e but did however not detect any differences at this time point.

## Conclusions

Overall, we show here that NSCLC cells surviving a short cisplatin pulse treatment have heterogeneous gene expression patterns. We identify a number of genes as potential markers of intrinsic cisplatin refractoriness, such as *DKK1*, *FMN1*, *XRCC2* and *LGALS9*. Moreover, we demonstrate that *DKK1* is a possible target that can be used for cisplatin sensitizing purposes in NSCLC cells and likely also other tumor types such as ovarian carcinoma. Our study therefore emphasize that further studies should be performed with respect to *DKK1* and its interactome to reveal how it can be used to sensitize NSCLC to platinum-based therapy, especially in an *in vivo* setting such as NSCLC patient-derived xenografts.

### Availability of supporting data

The data set supporting the results of this article is available in NCBI's Gene Expression Omnibus [11] repository and accessible through GEO Series accession number GSE48244 [http://www.ncbi.nlm.nih.gov/geo/query/acc.cgi?acc=GSE48244].

## Additional files

Additional file 1: **Hierarchical clustering was performed using Partek Genomics Suite v6.6.** Fold changes for genes in cisplatin-surviving compared to untreated U-1810 cells for the replicates R1, R2 and R3 were used, where red designates upregulated and blue downregulated genes. All genes which were up- or down-regulated over 1.5-fold in any replicate were included (for those regulated in more than one replicate, the additional redundant ones were removed). (TIFF 1678 kb)
Additional file 2: **A plasmid carrying FMN1 was transfected into NSCLC U-1810 cells for 24 h, for which Lipofectamine-only served as control.** After another 24 h cells were tested for FMN1 expression by western blot (A) or subjected to cisplatin treatment for 72 h after which cell viability was examined by MTT (B). (A) Representative blot for FMN1 expression in which β-tubulin served as loading control. (B) Cell survival after FMN1 overexpression in U-1810 cells, given relative to Lipofectamine-treated cells. Data shown is the mean ± SEM of three experiments. (TIFF 120 kb)
Additional file 3: **A549 cells were transfected with non-targeting (NT) or DKK1-specific siRNA (si1).** (A) Silencing of DKK1 mRNA expression was confirmed by q-RT-PCR. 18S rRNA was used as a loading control. ***; p < 0.005 vs NT control. (B) A549 cells were transfected with siRNA-DKK1 or NT siRNA and colony formation capacity was assayed 9 days after treatment or not with a short pulse of cisplatin (1 h, 10 μM). Clonogenic capacity relative to untreated, NT siRNA-transfected cells, *; *p* < 0.05 (C) Western blots showing p21WAF1/Cip1 and Bcl-2 in A549 cells 24 h after treatment with cisplatin (1 h, 10 μM), which was performed on reseeded cells after the 72 h-transfection with non-targeting control or siRNA-DKK1 (si1). GAPDH was used as a loading control. (TIFF 423 kb)
Additional file 4: **Retreatment of the pooled surviving U-1810-clones from a first round of clonogenic survival, where the first treatment is indicated for cisplatin-surviving (10 μM, Cisplatin as 1st) or untreated (Untreated as 1st) clones.** (A) The relative clonogenic survival in the first (1st) round is depicted in the first white bar, the grey bars are data from the retreated (2nd) experiment as described above, also using 10 μM cisplatin. (B) MTT cell viability data. Doses used for the retreatment were from 0.5-50 μM cisplatin and viability was analyzed after 72 h. Average ± SD from three experiments, MTT was performed in triplicate. (TIFF 54 kb)

## Abbreviations

AKT: v-Akt murine thymoma viral oncogene homolog
AUC: area under the curve
BAD: Bcl-2-associated death promoter
BAK: Bcl-2 homologous antagonist/killer
BAX: Bcl-2-associated X protein
Bcl-2: B-cell lymphoma 2
Bcl-xL: B-cell lymphoma-extra large
CDKN1A: cyclin-dependent kinase inhibitor 1A
DKK1: Dickkopf WNT signaling pathway inhibitor 1
DLG4: disks large homolog 4
DNAJB6: DnaJ homolog subfamily B member 6
EGFR: epidermal growth factor receptor
EPHB2: ephrin type-B receptor 2
ERCC1: excision repair cross-complementing rodent repair deficiency, complementation group 1
EZH2: enhancer of zeste homolog 2
FMN1: formin 1
GEO: Gene Expression Omnibus
GSK3B: glycogen synthase kinase 3 beta
HDAC2: histone deacetylase 2
ICL: inter-strand crosslink
IPA: Ingenuity Pathway Analysis tool
KRAS: Kirsten rat sarcoma viral oncogene homolog
LC: lung cancer
LGALS9: lectin, galactoside-binding, soluble 9
MAPK1: mitogen activated protein kinase 1
MAPT: microtubule-associated protein tau
MTT: 3-[4,5-dimethylthiazol-2-yl]-2,5-diphenyl-tetrazolium
NSCLC: non-small cell lung cancer
p21^WAF1/Cip1^: p21 wild-type activating fragment-1/cyclin-dependent kinase inhibitory protein-1
PI3K: phosphatidylinositol-3-kinase
PLIER: probe logarithmic intensity error estimation
PM GCBG: perfect match GC composition-based background correction
q-RT-PCR: quantitative real time polymerase chain reaction
RRM1: ribonucleotide reductase M1
SCLC: small cell lung cancer
SNORD: small nucleolar RNAs
TCF4: transcription factor 4
XRCC2: X-ray repair cross-complementing protein 2
