# Supplementary figures and images for: DKK1 is a potential novel mediator of cisplatin-refractoriness in non-small cell lung cancer cell lines
Source: BMC Cancer. 2015 Sep 9;15:628. doi: 10.1186/s12885-015-1635-9 (PMC4565013; doi:10.1186/s12885-015-1635-9)

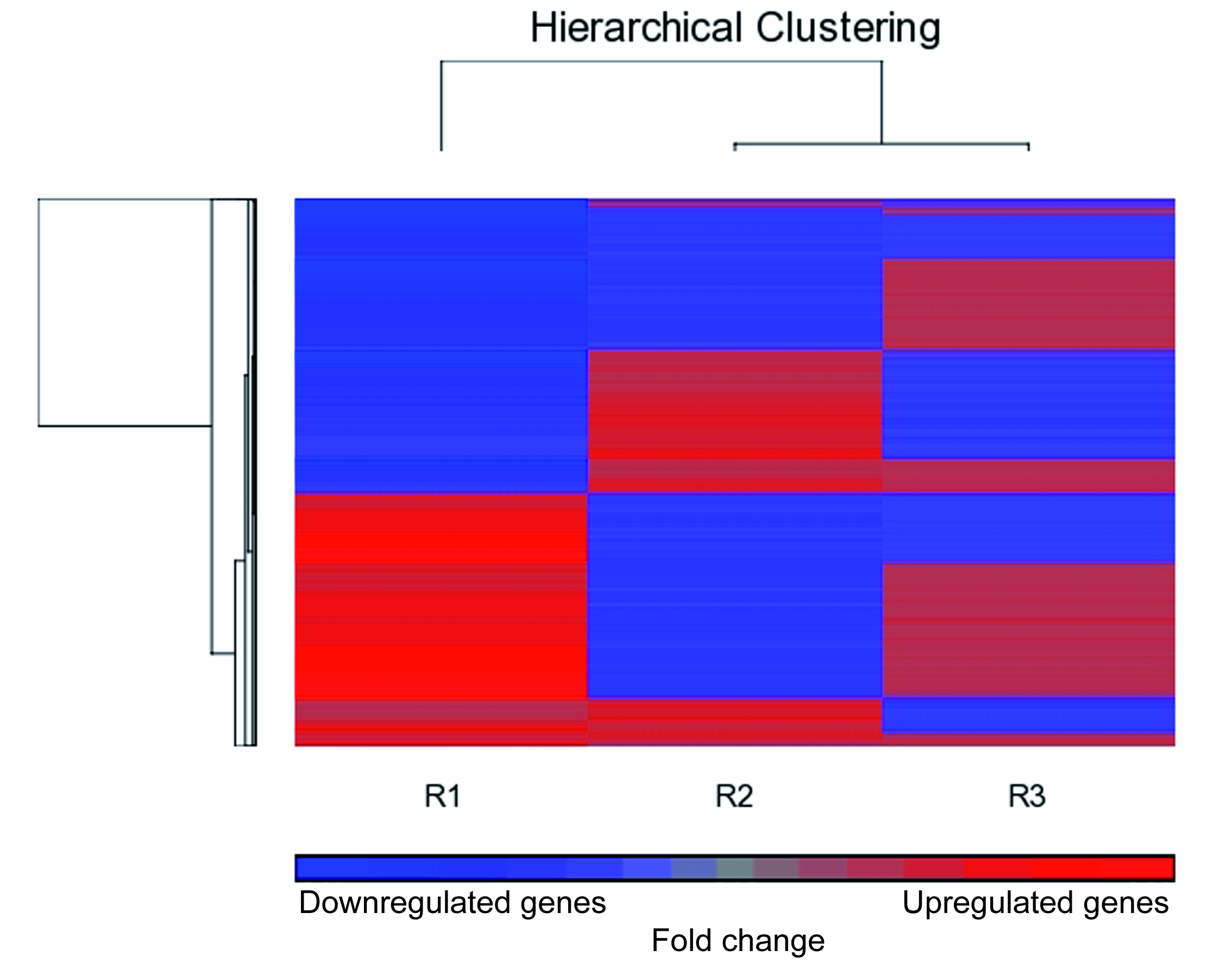

Supplement: Additional file 1: — Hierarchical clustering was performed using Partek Genomics Suite v6.6. Fold changes for genes in cisplatin-surviving compared to untreated U-1810 cells for the replicates R1, R2 and R3 were used, where red designates upregulated and blue downregulated genes. All genes which were up- or down-regulated over 1.5-fold in any replicate were included (for those regulated in more than one replicate, the additional redundant ones were removed). (TIFF 1678 kb) [file 12885_2015_1635_MOESM1_ESM.tiff]

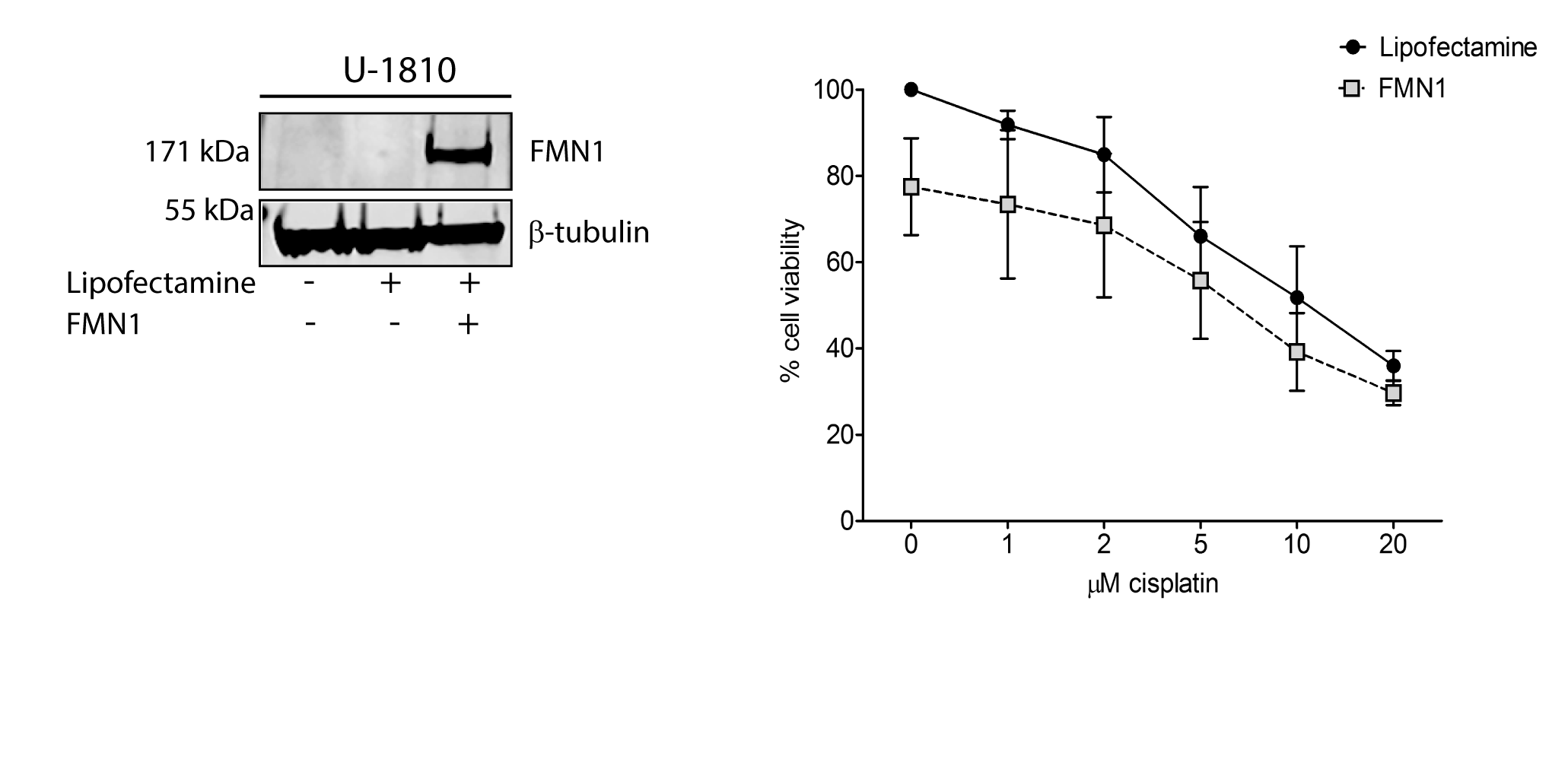

Supplement: Additional file 2: — A plasmid carrying FMN1 was transfected into NSCLC U-1810 cells for 24 h, for which Lipofectamine-only served as control. After another 24 h cells were tested for FMN1 expression by western blot (A) or subjected to cisplatin treatment for 72 h after which cell viability was examined by MTT (B). (A) Representative blot for FMN1 expression in which β-tubulin served as loading control. (B) Cell survival after FMN1 overexpression in U-1810 cells, given relative to Lipofectamine-treated cells. Data shown is the mean ± SEM of three experiments. (TIFF 120 kb) [file 12885_2015_1635_MOESM2_ESM.tiff]

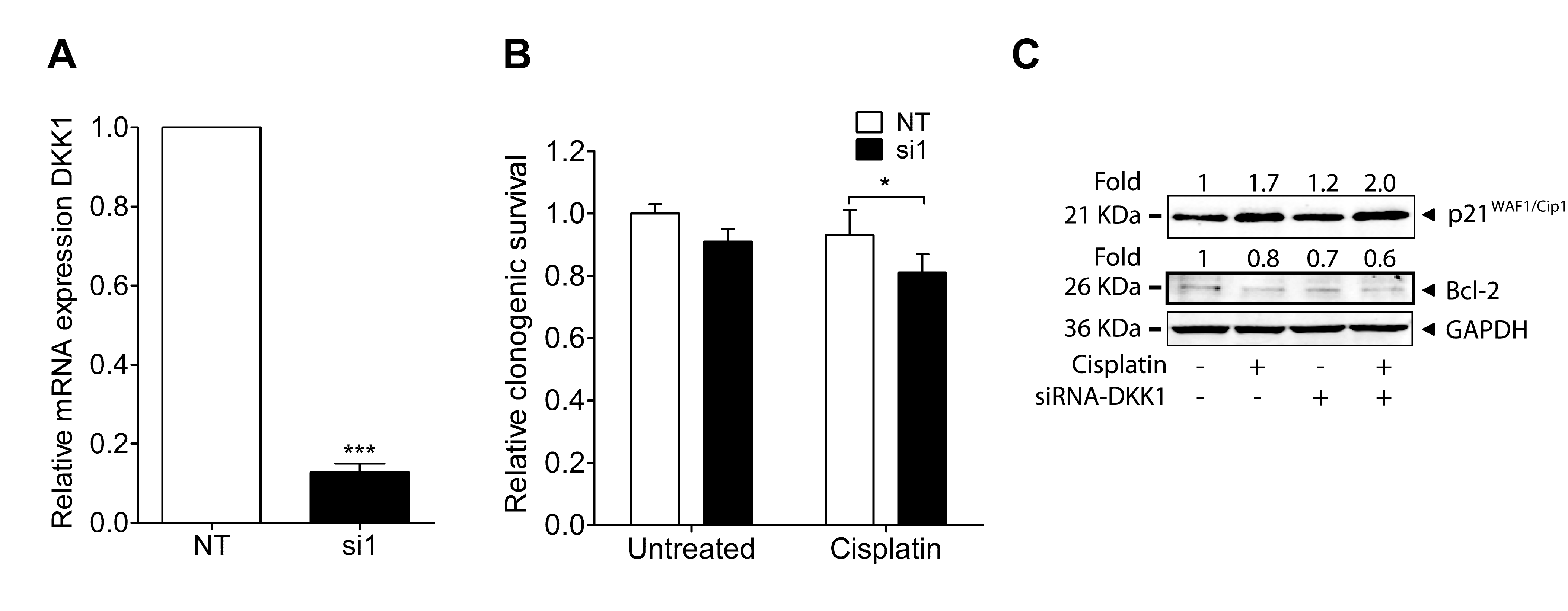

Supplement: Additional file 3: — A549 cells were transfected with non-targeting (NT) or DKK1-specific siRNA (si1). (A) Silencing of DKK1 mRNA expression was confirmed by q-RT-PCR. 18S rRNA was used as a loading control. ***; p < 0.005 vs NT control. (B) A549 cells were transfected with siRNA-DKK1 or NT siRNA and colony formation capacity was assayed 9 days after treatment or not with a short pulse of cisplatin (1 h, 10 μM). Clonogenic capacity relative to untreated, NT siRNA-transfected cells, *; p < 0.05 (C) Western blots showing p21WAF1/Cip1 and Bcl-2 in A549 cells 24 h after treatment with cisplatin (1 h, 10 μM), which was performed on reseeded cells after the 72 h-transfection with non-targeting control or siRNA-DKK1 (si1). GAPDH was used as a loading control. (TIFF 423 kb) [file 12885_2015_1635_MOESM3_ESM.tiff]
